# “Virtual” (Computed) Fractional Flow Reserve: Current Challenges and Limitations

**DOI:** 10.1016/j.jcin.2015.04.006

**Published:** 2015-07

**Authors:** Paul D. Morris, Frans N. van de Vosse, Patricia V. Lawford, D. Rodney Hose, Julian P. Gunn

**Affiliations:** ∗ Department of Cardiovascular Science, University of Sheffield, and Insigneo Institute for In Silico Medicine, Sheffield, United Kingdom; † Department of Cardiology, Sheffield Teaching Hospitals NHS Foundation Trust, Sheffield, United Kingdom; ‡ Department of Biomedical Engineering, Eindhoven University of Technology, Eindhoven, the Netherlands

**Keywords:** computational fluid dynamics, computational modeling, coronary angiography, fractional flow reserve, percutaneous coronary intervention, virtual fractional flow reserve, CAG, invasive coronary angiography, CFD, computational fluid dynamics, CTCA, computed tomographic coronary angiography, FDA, U.S. Food and Drug Administration, FFR, fractional flow reserve, PCI, percutaneous coronary intervention, QCA, quantitative coronary angiography, vFAI, virtual functional assessment index, vFFR, virtual fractional flow reserve, 3D, 3-dimensional

## Abstract

Fractional flow reserve (FFR) is the “gold standard” for assessing the physiological significance of coronary artery disease during invasive coronary angiography. FFR-guided percutaneous coronary intervention improves patient outcomes and reduces stent insertion and cost; yet, due to several practical and operator related factors, it is used in <10% of percutaneous coronary intervention procedures. Virtual fractional flow reserve (vFFR) is computed using coronary imaging and computational fluid dynamics modeling. vFFR has emerged as an attractive alternative to invasive FFR by delivering physiological assessment without the factors that limit the invasive technique. vFFR may offer further diagnostic and planning benefits, including virtual pullback and virtual stenting facilities. However, there are key challenges that need to be overcome before vFFR can be translated into routine clinical practice. These span a spectrum of scientific, logistic, commercial, and political areas. The method used to generate 3-dimensional geometric arterial models (segmentation) and selection of appropriate, patient-specific boundary conditions represent the primary scientific limitations. Many conflicting priorities and design features must be carefully considered for vFFR models to be sufficiently accurate, fast, and intuitive for physicians to use. Consistency is needed in how accuracy is defined and reported. Furthermore, appropriate regulatory and industry standards need to be in place, and cohesive approaches to intellectual property management, reimbursement, and clinician training are required. Assuming successful development continues in these key areas, vFFR is likely to become a desirable tool in the functional assessment of coronary artery disease.

Fractional flow reserve (FFR) is the accepted gold standard for assessing the physiological significance of coronary artery lesions during invasive coronary angiography (CAG) (1). FFR-guided percutaneous coronary intervention (PCI) improves patient outcomes, reduces stent insertions, and reduces costs (2). However, even in the United States and European Union, where FFR use is amongst the highest, FFR is used in <10% of PCI procedures and in fewer diagnostic cases 3, 4. Why is this so, 20 years after its introduction, with such a strong evidence base? First, decisions regarding the mode of revascularization are usually made at the time of invasive CAG, but this is limited specifically to PCI operators, working in an interventional catheter laboratory, with the time and facilities to perform FFR. Second, the procedure is prolonged, and short-term costs increased, because of the need for a pressure wire and a hyperemic drug. Third, many operators remain confident that their own visual assessment is physiologically accurate, allied to a misconception that multiple visual assessments (e.g., in a “Heart Team” setting) or a prior noninvasive test of ischemia improve their accuracy. Finally, despite the FAME (Fractional Flow Reserve Versus Angiography for Multivessel Evaluation) trial data 5, 6, some clinicians remain skeptical of the value of PCI in the context of stable coronary artery disease (7), which reduces enthusiasm for invasive FFR assessment.

## Virtual FFR

There is, therefore, a need for a method that delivers the benefits of physiological lesion assessment to every cardiologist without the practical drawbacks that limit the invasive technique. Several groups have used computational fluid dynamics (CFD) allied to anatomical models based upon coronary imaging to compute FFR without passage of a pressure wire. This is becoming known as virtual fractional flow reserve (vFFR) or computed FFR.

## Computational Fluid Dynamics

**Figure 1:**
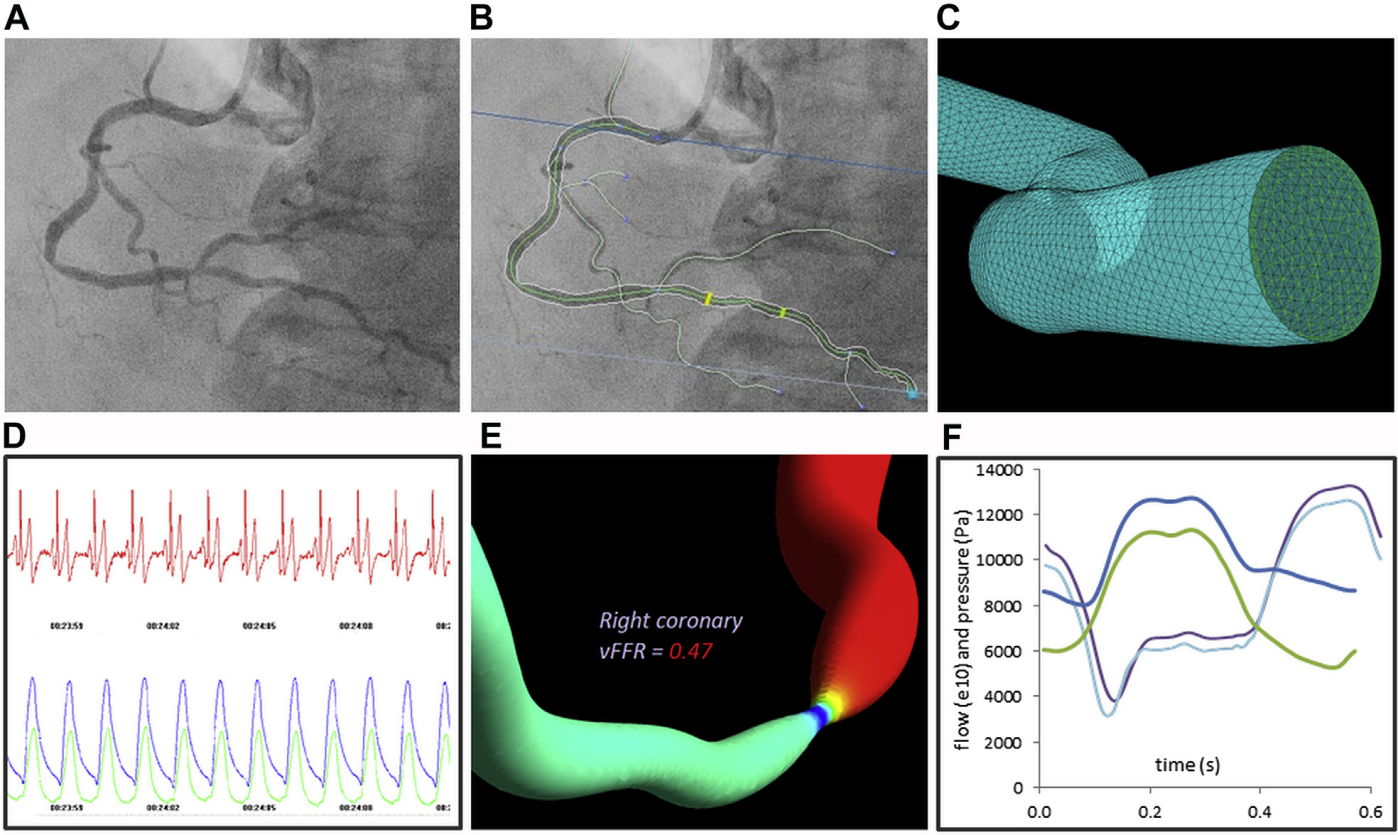
A vFFR Model Workflow Coronary angiogram **(A)** is “segmented” and reconstructed **(B)** into a 3-dimensional (3D) model **(C)**. Surface and volumetric meshing “discretize” the patient-specific geometry **(C)**. The physiological conditions beyond the modeled section must be represented at each boundary, that is, “boundary conditions” **(D)**. Computational fluid dynamics simulation computes the pressure gradient, using the anatomical 3D model “tuned” with physiological parameters. Pressure ratio is computed from output data **(E)**. Results are validated against invasive measurements during development **(F)**. vFFR = virtual fractional flow reserve.

CFD is a branch of fluid mechanics and a specialist area of mathematics that uses numerical methods and computer algorithms to simulate and analyze fluid flow. CFD modeling is a highly accurate method that is relied upon in a wide range of safety-critical industrial applications, such as aircraft design. Almost all CFD analyses are based upon solving the Navier Stokes and conservation equations: the governing equations of fluid flow, which represent Newton’s second law (balance of momentum) and conservation of mass, respectively, in a continuums approach. CFD models of vFFR require anatomical and physiological inputs. Arterial anatomy is “segmented” from coronary imaging (computed tomography [CT] or invasive angiography) and reconstructed into 2- or 3-dimensional (3D) in silico surface representations. These arterial models must then be discretized, or “meshed,” into a finite number of volumetric elements. In addition, the time-step of the simulation must be defined. The processes of spatial and temporal “discretization” determines the fidelity and refinement of a given analysis. The physical conditions at the inlet, outlet(s), and arterial walls must then be defined (i.e., the “boundary conditions”). A computer file that fully defines the spatial, temporal, and physiological bounds of the modeled system is then generated and executed using specialist software known as a CFD “solver,” which simulates the distribution and dynamics of blood pressure, flow, and shear stress within the artery over time. These data are used to generate predictions regarding pressure and flow changes across coronary stenoses, from which vFFR can be calculated at any point along the vessel. These processes and the key stages of developing a typical vFFR workflow are demonstrated in Figure 1.

Theoretically, computing the pressure drop across a stenosis is an elementary CFD problem. However, due to practical and clinical limitations, the geometric and hemodynamic factors that influence blood flow and energy loss along a diseased coronary artery are complex.

## vFFR Derived From Computed Tomographic Coronary Angiography

vFFR derived from computed tomographic coronary angiography (CTCA) (vFFR_CT_) has accumulated the most significant evidence base to date. DISCOVER-FLOW (Diagnosis of Ischemia-Causing Stenoses Obtained Via Noninvasive FFR) was the first major published trial of vFFR_CT_ (Table 1) (8). With measured FFR as the reference standard, this study of 103 patients (159 vessels) demonstrated how CTCA-derived vFFR added value to standard CTCA in reducing the number of false positive results. On a per-patient (cf., per-vessel) basis, vFFR_CT_ diagnosed physiological lesion significance (FFR >0.80 or ≤0.80) with 87.4% (95% confidence interval [CI]: 79% to 93%) overall accuracy. This was significantly superior to standard CTCA (61%; 95% CI: 51% to 71%). The results of the larger follow-up DEFACTO (Determination of Fractional Flow Reserve by Anatomic Computed Tomographic AngiOgraphy) trial (per-patient diagnostic accuracy 73%; 95% CI: 67% to 78%), however, did not meet the author’s pre-defined outcome goal in terms of per-patient diagnostic accuracy (9). The more recent HeartFlow NXT (HeartFlow Analysis of Coronary Blood Flow Using CT Angiography: Next Steps) trial further assessed vFFR_CT_, utilizing “updated proprietary software,” “improved segmentation,” “refined physiological models,” and “increased automation,” which generated improved diagnostic accuracy in a larger cohort of 251 patients (484 vessels) (per-patient accuracy 81% (95% CI: 76% to 85%) (10). Subsequently, HeartFlow, Inc. has gained U.S. Food and Drug Administration (FDA) approval for the use of vFFR_CT_ as a class II Coronary Physiologic Simulation Software Device (11). vFFR_CT_ is computed using standard CTCA protocols without induction of hyperemia and is, therefore, a highly practical and useful method in outpatients, which may reduce the number of unnecessary referrals for invasive angiography. However, only dichotomized data have been reported thus far. Data regarding the agreeability between vFFR and measured values were not published in the aforementioned trials. The vFFR_CT_ method assumes a predictable response to adenosine, which is controversial (12). Furthermore, vFFR_CT_ is limited by the same factors that limit standard CTCA, namely calcification, tachycardia, and arrhythmia. Although the latest technological developments used in the NXT trial clearly improved accuracy in this cohort, a precise description of the methods and algorithms have not been published, which precludes further scrutiny.

## vFFR From Invasive Angiography

All patients considered for revascularization undergo invasive coronary angiography (CAG), which remains the gold standard method for diagnosing and assessing coronary artery disease. Several groups have, therefore, attempted to model vFFR based upon CAG imaging (vFFR_CAG_). In the VIRTU-1 (VIRTUal Fractional Flow Reserve From Coronary Angiography) study, Morris et al. (13) constructed a computational workflow that computed vFFR from CAG images. In 35 diseased vessels, the VIRTUHEART model (University of Sheffield, Sheffield, United Kingdom) predicted (dichotomized) physiological lesion significance with 97% accuracy, albeit with a paucity of FFR cases within the critical 0.75 to 0.85 range. Average error between vFFR and measured FFR was ±0.06.

Tu et al. (14) developed a model based upon 3D quantitative coronary angiography (QCA) and the much faster steady-flow CFD analysis (cf., transient, see the following text), with mean hyperemic flow velocity derived from Thrombolysis In Myocardial Infarction (TIMI) frame counting. Unlike the aforementioned models, this model requires induction of hyperemia (a potential factor contributing to current FFR underuse) during CAG. Nevertheless, their model provided diagnostic accuracy of 88.3% in 77 cases.

Papafaklis et al. (15) developed a model predicting “virtual functional assessment index (vFAI) for fast functional assessment of intermediate coronary lesions.” This model does not compute vFFR. Instead, this model uses 3D QCA and steady-flow CFD analysis to compute the ratio of distal to proximal pressure over the lesion for flows in the range 0 to 4 ml/s, normalized by the ratio over this range for a normal artery. The pressure ratio as a function of flow is described as a quadratic equation, with coefficients determined by steady-state CFD analysis at 2 flow rates (1 and 3 ml/s). vFAI is numerically equal to the average of the computed pressure ratio over this flow range. This approach is fast, but it ignores the critical physiological influence of the coronary microvascular resistance (see the following text). In their trial of 139 vessels (n = 120), vFAI was superior to standard 3D QCA in predicting physiological lesion significance; a vFAI cut-off of ≤0.82 was associated with overall diagnostic accuracy of 88%.

## Advantages of vFFR

**Figure 2:**
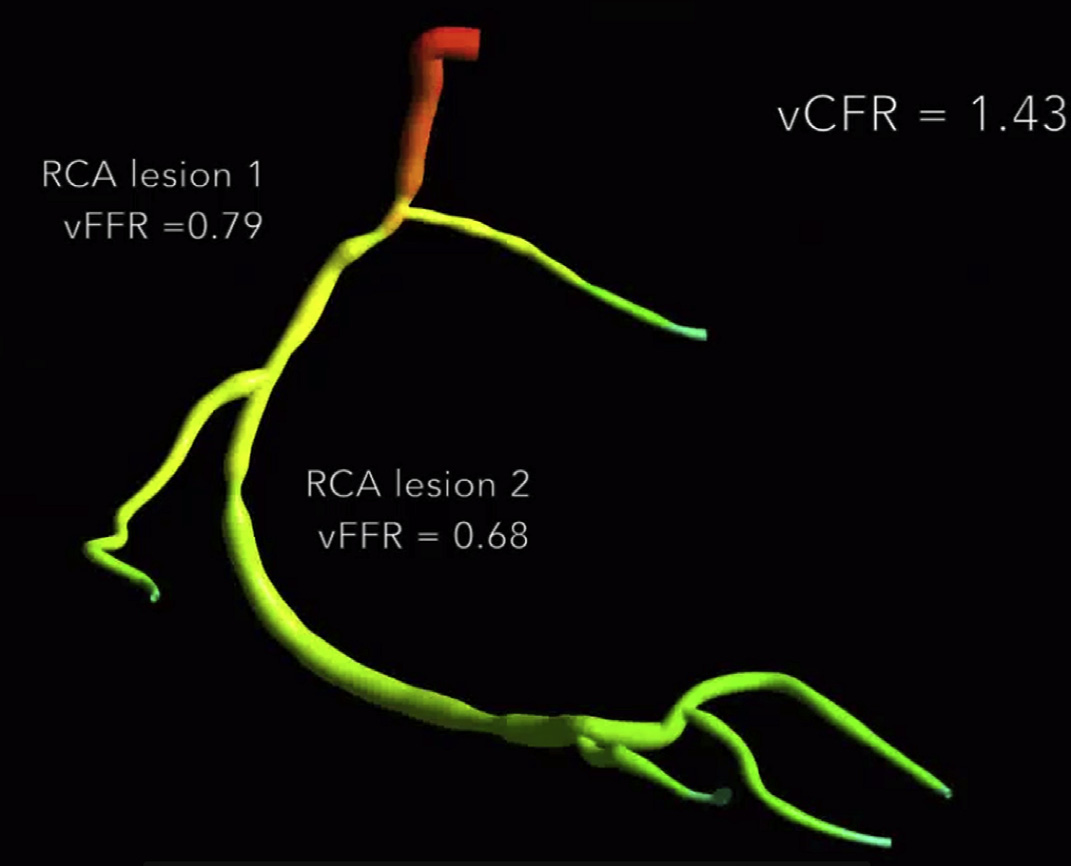
vFFR Virtual Pullback Result Pressure distribution throughout a right coronary artery (RCA) allowing individual lesion evaluation. vCFR = virtual coronary flow reserve; vFFR = virtual fractional flow reserve.

Early vFFR results using CT (16) and angiographic images 13, 14 are encouraging, and in the case of CTCA vFFR, have reached clinical applicability. Beyond simply replacing invasive FFR and providing the benefits of physiological assessment in the many patients not currently afforded them, vFFR offers some additional advantages. vFFR provides a virtual pullback, in which pressure is demonstrated at all points within a branched coronary arterial tree in a single analysis, allowing the physiological significance of serial lesions to be evaluated accurately and individually, a process far more challenging with invasive FFR (Figure 2). It can also provide a “virtual stenting” facility, whereby the physiological effect of alternative interventional strategies can be trialed in silico (by computer simulation) before treatment is delivered in vivo. vFFR can also assess any segment of the coronary tree, including those to which it might be challenging to pass a pressure wire.

## Challenges

Several scientific, logistic, and commercial challenges must be overcome, however, before vFFR can enter routine clinical use.

## Segmentation

Segmentation from medical images, whether they be derived from CTCA or CAG, is crucial to the accuracy of CFD simulation. CT-based vFFR is apposite for truly noninvasive vFFR, but CTCA is mainly used to rule out coronary artery disease in those with low-to-medium pre-test probability of coronary artery disease, rather than for detailed planning of revascularization. In many patients, CTCA does not provide sufficient image quality for accurate segmentation, whether due to cardiac or respiratory movement, tachycardia or arrhythmia leading to a “stair-step” artefact, phase misregistration or blurring, or calcification leading to “blooming” or “streaking” (17). The noninvasive nature of CTCA also prevents the measurement of other physiological parameters that can be used to inform CFD simulation. Segmentation from CAG is also challenging. Current protocols most commonly segment from just 2 projections, which may under-represent the full 3D anatomy. Software is used to correct for patient movement between acquisitions, but this is not entirely robust, and biplane CAG acquisition is not available in the majority of catheter laboratories. Rotational coronary angiography can be successful because it offers the potential to use multiple views in the reconstruction, or at least the option to select 2 optimal projections, eliminating vessel overlap, foreshortening, and inadequate opacification, but is also not widely available. Methods such as intravascular ultrasound and (particularly) optical coherence tomography would add detailed anatomical accuracy, but problems of image co-registration, the techniques’ invasiveness, and increased cost on top of the CTCA and CAG imaging, make them impractical in the context of vFFR.

## Boundary Conditions

The physical conditions affecting each of the boundaries (inlets, outlets, and vessel walls) must be accurately represented in the model. Identifying a noninvasive strategy for tuning (or “parameterizing”) these boundary conditions probably presents the greatest challenge. For the purpose of computing vFFR, there is no strong evidence that it is necessary to represent the motion or compliance of the vessel wall. The inlet pressure is relatively easy to determine, either from direct measurement of aortic pressure in a CAG-based model, or from cuff-pressure with a transfer function in the case of CTCA.

**Figure 3:**
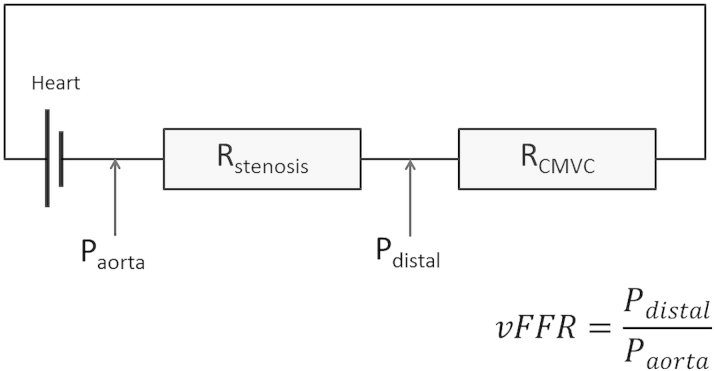
Electrical Model Demonstrating the Importance of the Distal Boundary During vFFR Computation Pressure (P), flow, and resistance (R) are analogous to electrical potential difference, current, and resistance. R_stenosis_ and coronary microvasculature circulation resistance (R_CMVC_) are effectively 2 resistors arranged in series. Therefore, even if P_aorta_ and R_stenosis_ are known (or computed), computation of virtual fractional flow reserve (vFFR) is wholly dependent on the parameters of R_CMVC,_ because this determines P_distal_ and, hence, vFFR. If CMVC resistance is overestimated, lesion severity and the potential benefit from revascularization are underestimated, that is, vFFR > fractional flow reserve, and vice versa.

The distal outlet boundary conditions are difficult to determine because they are those of the coronary microvasculature circulation (CMVC) that are heterogeneous in health and disease and require direct invasive measurement, which we wish to avoid in minimally invasive modeling. Figure 3 demonstrates why the accuracy of vFFR computation is absolutely dependent upon the CMVC.

This is relevant when considering the vFAI, which is entirely a function of stenosis geometry and ignores the influence of the CMVC (15). Although likely to be superior to QCA alone (because the geometric description is transformed into a more physiologically relevant measure, namely pressure ratio), vFAI cannot be a substitute for FFR, because unlike FFR, it ignores the CMVC. The vFAI will always be low, indicating the need for intervention, if the lesion is geometrically significant, whereas the FFR might be high or low for the same lesion depending on the overall physiology, and in particular on the status of the coronary microvasculature. Although on average there is expected to be a correlation between vFAI and FFR (because the pressure ratio is likely to be lower when the lesion is more geometrically significant), differences would occur whenever the impedance of the microvasculature deviates significantly from normal, and these are exactly the circumstances that are captured by FFR.

**Figure 4:**
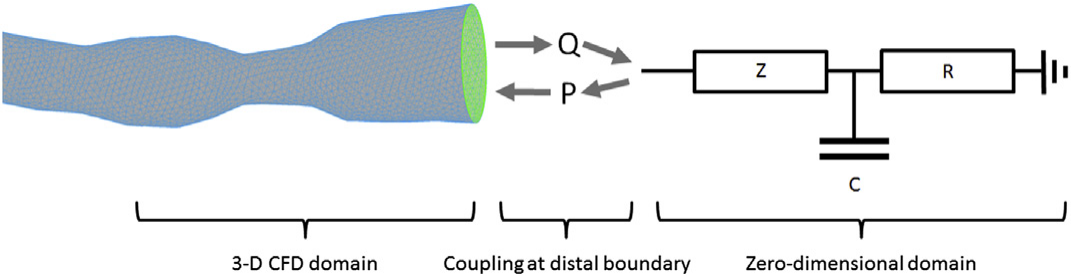
Modeling the distal coronary boundary The 3-dimensional (3D) coronary model is coupled to a 0-dimensional, lumped parameter model to represent the physiological conditions at the distal boundary **(right)**. An electrically analogous Windkessel design represents the impedance (Z), resistance (R), and capacitance (C) of the distal coronary microvasculature circulation. The algebraically coded Windkessel computes pressure (P) and flow (Q), which dynamically informs the 3D computational fluid dynamics simulation. As in Figure 3, model parameters are vital to patient-specific accuracy.

Morris et al. (13) and Taylor et al. (17) both apply a lower-order model to represent the distal (outlet) boundaries (Figure 4). Morris et al. (13) employed a generic, average, “one-size-fits-all” approach to parameterization. Although this model generated impressive overall accuracy, individual patient accuracy was improved significantly (the error was halved) by applying invasively measured parameters of CVMC (13). Taylor et al. (17) implement a strategy based upon ventricular wall volume (from CT imaging), brachial blood pressure, and a morphometric law that defines the relationship among resistance, flow, and vessel diameter. Although this is a more sophisticated strategy, hyperemic CMVC resistance is similarly derived from averaged, population-based data. Ultimately, both models are prone to errors secondary to variability in hyperemic CMVC resistance (Figure 3).

Applying flow as a boundary condition is a sensible alternative, because this accounts for CMVC resistance. However, measurement of absolute hyperemic coronary flow is invasive, challenging, and requires induction of hyperemia. Estimating mean flow rate from TIMI frame count yields respectable results that correlate to Doppler wire analysis, but this is only apposite for steady-flow analysis and cannot easily be applied to a transient CFD analysis (see the following text) (14).

If vFFR is to succeed, a strategy that provides personalized estimation of the distal boundary conditions, using noninvasive clinical data, is required.

## CFD Simulation

The optimal method of CFD simulation is also yet to be determined. The coronary circulation is a dynamic, 3D, pulsatile system; transient (timed dependent) 3D CFD simulation is, therefore, considered the most representative and accurate method. This involves solving for millions of degrees of freedom (the possible independent variations in a dynamic system within the constraints imposed upon it) of a nonlinear, partial differential equation simultaneously and repeatedly, hundreds or thousands of times each cardiac cycle. This is computationally time-consuming (approximately 12 to 24 h), notwithstanding the steady increase in computational speed. Modelers have sought quicker alternatives.

Steady-state CFD analysis can be used rather than transient. This runs more quickly (2 min) but does not represent the accelerative, time-dependent behavior of pulsatile blood flow. However, this has been demonstrated to yield respectable vFFR accuracy (14). Reduced order (1-dimensional) CFD simulation is also fast, but does not represent the 3D nature of the coronary tree. Although steady flow analyses have simulated similar flow and shear stress patterns (18), the trade-off between simplifying computation and effect on accuracy needs to be fully evaluated.

## Computation Time

Prolonged vFFR computation times, over many hours, have been a major concern and may limit vFFR applicability. This is less crucial for CT-FFR, but is more important for CAG-related methods where real-time, on-table results are required. Off-line, remote, supercomputer simulation represents one solution, but this is less attractive than system acceleration that allows real-time processing within software integrated into local catheter-laboratory systems. The adoption of very high-powered computation locally is not easily accommodated within parsimonious health care systems. Steady-state or reduced-order modeling are further options, with CFD results being generated within 5 min, which is comparable to invasive measurement (14). Unlike invasive FFR, vFFR can also evaluate several coronaries and lesions in a single analysis. Using steady-state or 1-dimensional modeling is attractive but may sacrifice accuracy. However, the challenge of system acceleration appears eminently achievable.

## Model Complexity and Design

Complex models, requiring invasive measurements with prolonged run times, have improved accuracy, but physicians require simple, rapid systems of sufficient accuracy to inform treatment decisions. Further work is needed to discern models that balance these needs optimally. One potential solution is a multitiered approach that delivers fast results but with wide confidence margins, reserving more complex modeling for more borderline cases where increased precision is required. Furthermore, is there an appetite for remote supercomputation (raising issues of transferring large confidential datasets outside of the hospital), or would physicians prefer to run analyses themselves using systems within their catheterization laboratory?

## Accuracy and Validation

Accuracy is absolutely key to vFFR’s success. However, what constitutes accuracy is yet to be defined. Perhaps most important is whether vFFR correctly assigns a patient to treatment to produce alleviation of symptoms. Currently, this accords with a treatment threshold of FFR <0.80. A related measure of accuracy is how close vFFR values approximate the measured values over the whole range, and if so, how close is acceptable? Because FFR itself varies between repeat measurements (19), the comparative accuracy of vFFR is also restricted. Furthermore, should accuracy be defined on a per-patient or per-vessel basis? A Bland-Altman plot is the best statistical metric for evaluating vFFR accuracy against FFR, but this method does not lend itself to making comparison between different models. Although often quoted, correlation coefficients are misleading and do not reflect agreeability. Ultimately, demonstrating clinical success is vital, regardless of agreement with other methods, and prospective, randomized controlled trials will be necessary. If computed clinical results are to be truly believed and used in making clinical decisions, then, in addition to comparing computed and measured results, authors should describe their numerical methodology and demonstrate validation of their algorithms. Although CFD software packages can be used by the nonexpert, considerable multidisciplinary experience is required to generate robust and reliable data. Successful validation requires clinical, mathematical, biomechanical engineering, and modeling expertise.

## Commercial Considerations

Around 1 million PCI procedures are performed annually in the United States at a cost of approximately $10 billion; the rewards for successfully modeling vFFR are therefore considerable. Understandably, academics, funders, and investors are keen to protect their intellectual and financial investment and to prevent their own work being restricted by another group or interested party. CFD is a mature technology with a significant base of published data (“prior art”), but there are several patentable aspects of the analysis process, and a number of patents have already been granted.

In the context of interventional cardiology, CFD modeling is a new technology that is potentially disruptive, especially to manufacturers of hardware that may become redundant if vFFR is successful. Traditionally-minded manufacturers and physicians will need to embrace these new techniques and engage with academics and modelers to ensure that the best methods are adopted.

Medicare reimbursement has boosted FFR use in the United States. Now that CTCA-based vFFR has been approved by the FDA, a similar arrangement is needed.

There are currently no industry standards regarding accuracy, reliability, or validation. The FDA is addressing this through a benchmarking initiative that aims to advance the application of CFD technology within the regulatory context (20). A consistent, evidence-based strategy administered by experts is also needed in Europe.

## Trial Evidence and Clinical Considerations

Most physicians are unaware of the basic principles, strengths, and weaknesses of CFD modeling and, hence, vFFR. Academics, modelers, and engineers should engage with the clinical community in an open and transparent manner with regard to the merits and limitations of computational modeling. vFFR systems must be simple and intuitive to maximize adoption and for safe use by physicians who are not CFD experts. Prior to clinical acceptance, 2 imperatives must be met. The first is to demonstrate equivalence of vFFR to invasive FFR in clinical practice in situations when FFR is currently used. The second is to compare vFFR tools within traditional decision pathways at the stage of diagnostic angiography (CAG or CTCA) for the many thousands of patients who are not currently offered physiological assessment.

## Conclusions

A number of scientific, logistical, political, and commercial challenges must be overcome before vFFR enters routine clinical practice. Issues regarding model personalization, image segmentation, and boundary condition tuning are particularly important. These challenges are not insurmountable; early results are encouraging despite current limitations. Assuming successful development continues in these key areas, vFFR is likely to become a desirable tool in the functional assessment of coronary artery disease. CT derived vFFR is emerging as a useful tool for low /medium risk patients whereas more invasive vFFR applications are emerging as useful tools in higher risk patients undergoing invasive management.

## Figures and Tables

**Table 1:** Summary of Methodology and Precision of Models of vFFR

vFFR Model/Study (Ref. #)	Patients, Vessels (n)	Imaging Modality	CFD Simulation	Boundary Condition Strategy	Other	Versus Invasively Measured FFR					Approximate Run Time
Overall Diagnostic Accuracy∗	Area Under ROC Curve∗	Agreeability	Pearson Correlation Coefficient	Bias (Mean ± SD)
CTCA-FFR (16) DISCOVER-FLOW (8)	103, 159	CTCA	3D CFD	On the basis of vessel diameter and myocardial mass	Coupled lumped parameter model at outlets (HeartFlow software version 1.0)	84% (per-vessel)	0.90	Not reported quantitatively; see Bland-Altman plot in original publication	0.68 (p < 0.0001)	0.02 ± 0.116	Remote core laboratory computation
CTCA-FFR DeFACTO (9)	252, 407	CTCA	3D CFD	On the basis of vessel diameter and myocardial mass	Coupled lumped parameter model at outlets (HeartFlow software version 1.2)	69% (per-vessel)	0.81 (per-patient, per-vessel not reported)	Not reported quantitatively	0.63	0.06 (SD not reported)	Remote core laboratory computation
VIRTU-1 (13)	20, 35	CAG	Transient 3D	Pressure	Coupled lumped parameter model at outlets	97% (per-vessel)	0.97	vFFR deviated from mFFR by ± 0.06 (mean error) (Plus see Bland-Altman plot in original publication)	0.84	0.02 ± 0.080	12–24 h
vFAI (17)	120, 139	CAG	Steady-state 3D	Flow	Derived from ΔP-flow curve	87.8%	0.92	Not applicable†	0.78	0.00 ± 0.085	7 min
CTCA-FFR HeartFlow NXT (10)	251, 484	CTCA	3D CFD	On the basis of vessel diameter and myocardial mass	Coupled lumped parameter model at outlets (HeartFlow software version 1.4)	86% (per-vessel)	0.93 (per vessel)	Not reported quantitatively; see Bland-Altman plot in original publication	0.82	0.02 ± 0.074	Remote core laboratory computation
FFR_QCA_(14)	68, 77	CAG	Steady-state 3D	Mean flow (derived from TIMI frame count)		88.3%	0.93	Not reported quantitatively; see Bland-Altman plot in original publication	0.81	0.00 ± 0.06	5 min

Data derived on the basis of our best interpretation of the published data.

3D = 3-dimensional; CAG = invasive coronary angiography; CFD = computational fluid dynamics; CTCA = computed tomographic coronary angiography; DeFACTO = Determination of Fractional Flow Reserve by Anatomic Computed Tomographic AngiOgraphy; DISCOVER-FLOW = Diagnosis of Ischemia-Causing Stenoses Obtained Via Noninvasive FFR; FFR = fractional flow reserve; mFFR = invasively measured fractional flow reserve; HeartFlow NXT = HeartFlow Analysis of Coronary Blood Flow Using CT Angiography: Next Steps; QCA = quantitative coronary angiography; ROC = receiver-operating characteristic; TIMI = Thrombolysis In Myocardial Infarction; vFAI = virtual functional assessment index; vFFR = virtual fractional flow reserve; VIRTU-1 = VIRTUal Fractional Flow Reserve From Coronary Angiography.

∗ Diagnostic accuracy in diagnosing physiological lesion significance of an invasively measured FFR of <0.80 or >0.80.

† vFAI is a surrogate marker of functional lesion assessment.
